# The Week's Work

**Published:** 1911-06-24

**Authors:** 


### The Week's Work.

### A SANATORIUM'S APPEAL TO MR. LLOYD GEORGE.

Before coming to its present decision to remove to another site, the Royal National Sanatorium for Consumption and Diseases of the Chest at Bournemouth asked the Chancellor of the Exchequer whether it could expect assistance. In his reply, Mr. Lloyd George said that he was unable to express an opinion on individual cases until the Insurance Bill had been passed. This answer probably was anticipated by the Bournemouth Governors, who, so far as we know, have been the only sanatorium authorities to show much public and practical interest in the new condition which the Bill proposes to create.

### THE BRITISH HOSPITALS ASSOCIATION CONFERENCE SUPPORTED IN BIRMINGHAM.

We publish on another page a report of the meeting of fifty Midland Hospitals at Birmingham on Wednesday last week to discuss the Insurance Bill. Mr. Neville Chamberlain, of the General Hospital, presided. He recalled the fact that Mr. Howard Collins, house governor of the Birmingham General Hospital, had been placed on the Committee formed by the British Hospitals Association to wait upon the Chancellor. As Mr. Collins was the only representative of the Midland Hospitals upon it, Mr. Chamberlain thought that his words would carry more weight if he could speak as their informed representative; hence the meeting. Mr. Chamberlain then quoted the Chancellor's remark to the British Medical Association that: "The great curative institutions are voluntary institutions in every sense of the term, and we don't propose to exercise any control over them. What we propose is that the health committees and the approved societies shall have power to subscribe to these institutions." But where, asked Mr. Chamberlain, was the clause empowering the health committees to subscribe? Mr. Howard Collins produced figures to show that in the case of thirty-two hospitals within fifty miles of Birmingham some £100,000 was affected, directly in the case of employers' and Saturday fund contributions, and indirectly in the case of the Sunday fund and general subscriptions, out of a total income of some £194,000. Then Mr. Walter A. Crosbee, Chairman of the Queen's Hospital, read his own institution's recommendations, which we print in full in our report, to the effect that the treatment of inmates should be paid for. Mr. H. K. Beale, Chairman of the Children's Hospital, Birmingham, remarked that in treating uninsured persons the hospitals had an answer to those who were afraid of subscribing twice. Mr. William Peach, Chairman of the House Committee of Staffordshire General Infirmary, struck an optimistic note. He did not think it was the subscribers to the Saturday Fund so much as the very poor who found their way to hospitals. Mr. C. Perks and Mr. S. G. Dunn held opposite opinions as to the Bill's probable effect on the Birmingham General Dispensary, Mr. Dunn fearing the disaster that Mr. Perks did not foresee. The Rev. H. V. Stuart, of the North Staffordshire Infirmary, said that a contribution from the State must lead to the payment of the doctors for their services. Mr. J. C. Vaudrey, of the Birmingham Ear and Throat Hospital, called for the amendment of the Bill, and Mr. Frank Hancock, Secretary of the West Bromwich Hospital, feared for two-thirds of its income, whereupon a vigorous opposition to the Bill was urged by Mr. T. Foley Bache, honorary secretary to the same hospital. A resolution calling for compensation for loss of income, for the preservation of the independence of the voluntary hospitals, and for the preservation of their distinction from the Poor Law infirmaries was passed unanimously. In short the wisdom of the British Hospital Association in appointing a committee to wait on Mr. Lloyd George was fully borne out by the discussion, which we congratulate the Birmingham General Hospital on having initiated.

### THE NEW SITE FOR THE CHELSEA HOSPITAL.

The acre and a quarter which Lord Cadogan has just given to the Chelsea Hospital alters the whole situation which we noted on May 6 last. The problem whether to rebuild the whole hospital, or to provide a separate out-patient department and nurses' home is now brought to a pleasantly acute stage. Will those in favour of extension merely use the contiguity of the new land to the present buildings as an argument, or will Mr. Bland Sutton, who believes in a rebuilt hospital, carry the day? Probably the result will depend upon the estimated cost of a new hospital. The Council also will have to take into consideration the generally expected shrinking of out-patient work if the Insurance Bill passes into law. It will have to remember the probable increase of its number of in-patients, with its corollary of an increased number of beds and an increased staff of nurses. These external factors must be neglected no more than the internal ones of which the Council is intimately aware. A great responsibility rests upon those hospital authorities who are considering building schemes at the present time. In any case we may be sure that the unfortunate old system of housing the nursing staff under four roofs will be done away. We should not be surprised if an extension of the hospital were built upon the ground that Lord Cadogan has given.

### NEWSPAPER PROMPTITUDE.

The newspapers announce that the Royal South Hants and Southampton Hospital is to have a new out-patient department, a gift from Miss Burrell, costing over £10,000. This fact was made known in The Hospital six months ago, all that has happened since then being that Miss Burrell has increased her contribution from £7,000 to the larger sum. Probably, with her well-known local generosity, Miss Burrell from the first undertook the whole cost of the department, the original estimate of which has been exceeded.

### MR. THIES' SUCCESSOR.

We announced last week the appointment of Mr. Reginald R. Garratt to the secretaryship of the Royal Free Hospital, Gray's Inn Road, in the place of Mr. Conrad Thies, whose resignation takes effect, we believe, next month. We are now in a position to add some further particulars of Mr. Garratt's institutional and general career. After being educated at Dulwich College, Mr. Garrett spent three years studying surveying and land agency, and subsequently gained financial experience in the Union Bank of London. He then travelled, and in 1900 became associated with certain philanthropic institutions, more particularly with the work of Queen Victoria's Jubilee Institute for Nurses. Since 1900 he has, with the honorary treasurer, been responsible for adding to its capital funds, besides raising annually from £5,000 to £7,000 for its maintenance. During the same time he acted for three years as secretary to the Infants' Hospital. When the secretaryship to the Great Ormond Street Hospital was vacant Mr. Garratt was one of the three finally selected candidates.

### CHILDREN'S HOMES AND OUT-PATIENT DEPARTMENTS.

The *Child's Guardian*, the official organ of the N.S.P.C.C., announces that as the result of the generosity of a subscriber to the Society's funds a cottage home for the reception of six children is to be provided in a healthy district where the subscriber can herself personally supervise the arrangements for their comfort. The home is not intended to be a permanent residence, but will be used solely for the purpose of relieving children whose condition specially requires attention. Such private homes are already in existence in several parts of the country, and have proved of the greatest service to hospitals, especially to the out-patient departments, whose children, as a rule, cannot, owing to the limited supply of beds at the convalescent homes, be sent to the regular sick and convalescent institutes. There is scarcely a better object which private munificence can support than the establishment and maintenance of such homes for children who need fresh air, cleanliness, and hygienic care.

### THE CHOICE OF A HOSPITAL COMMITTEEMAN.

Not long ago we published a note on "The choice of a hospital secretary," which provoked some discussion, one secretary, for instance, writing that the post could be held adequately only by "a very exceptional fellow." The Inspector-General of Hospitals in New Zealand in his annual report, now gives some very pertinent hints to hospital committeemen, which are worth transcription: "An officer is only worth keeping" (Dr. Valentine says) "so long as he knows that he has something to learn. The 'indispensable' officer does not exist—at any rate, no institution can afford to retain him. . . . No man is fit to sit on a board who quotes what he has 'heard from a lady friend!'" Are we to suppose that the latter sort flourishes more easily where ladies are to be found on committees?

### A NEW POST FOR COLONEL DONEGAN.

As we go to press we learn that Lieutenant-Colonel J. F. Donegan, R.A.M.C., of Parkhurst Military Hospital, Isle of Wight, has been appointed to Woolwich for duty as Recruiting Medical Officer. The Press did not fail to note that the order relieving him of his command at Parkhurst was cancelled.

### A LONDON DISPENSARY'S LEAFLETS.

The success which has attended the system of dispensary consultations for poor mothers on the subject of their children's health has more than once been the occasion of comment in these columns. As far as London is concerned, the system has been more fully developed in Marylebone than elsewhere, perhaps because the St. Marylebone General Dispensary in Welbeck Street is able to command the services of the skilled experts from the neighbouring quarter. The importance of these attempts to prevent infant sickness and disablement is, from a national point of view, tremendous, and needs no emphasis in a professional journal. Although verbal advice is much more likely to be remembered, and can be made much more impressive than the written word can ever be, still in this sort of work there is really not time to go into every point of child hygiene personally with each patient, and leaflets are always found in the long run to be essential. The staff of the dispensary just mentioned has prepared an unusually full set of leaflets, which are well worth study by anyone who is interested in any similar institution. They are all couched in language expressly designed for the comprehension of the uneducated, and they embody only those rules of health which are not the ephemeral fads of the moment, but have been proved by long experience to be the proper way of life. The subjects of these brief pamphlets are Fresh Air and Ventilation; the Feeding of Infants; the Management of Children from one to five years of age; the Care of Children of School Age; Hints to Patients suffering from Indigestion; How to Prevent or Arrest Consumption; and Advice on the Care of the Teeth. In the whole of this series we have not found one single statement to which exception can be taken; and if the poor of Marylebone do but follow out the advice thus proffered them, it is indubitable that the next generation there will enjoy considerably better health and vigour than the last.

### THIS WEEK'S DRUG MARKET.

Business in the drug market continues quiet; a small business has been done, however, in a few articles. The demand for citric acid, tartaric acid, and cream of tartar continues good and prices are more likely to advance than recede. Quicksilver is slightly dearer. Cardamoms are somewhat cheaper. High prices are asked for ipecacuanha. Chamomiles are tending lower in value. Business has been done in ergot of rye at a high figure. Cod-liver oil is still tending rather lower in price. Low prices are quoted for chloral hydrate, and business has been done in rhubarb at slightly lower rates. Damiana leaves are somewhat cheaper.

